# Origins of amino acid transporter loci in trypanosomatid parasites

**DOI:** 10.1186/1471-2148-7-26

**Published:** 2007-02-23

**Authors:** Andrew P Jackson

**Affiliations:** 1 Wellcome Trust Sanger Institute, Wellcome Trust Genome Campus, Hinxton, Cambridgeshire. CB10 1SA. UK

## Abstract

**Background:**

Large amino acid transporter gene families were identified from the genome sequences of three parasitic protists, *Trypanosoma brucei, Trypanosoma cruzi *and *Leishmania major*. These genes encode molecular sensors of the external host environment for trypanosomatid cells and are crucial to modulation of gene expression as the parasite passes through different life stages. This study provides a comprehensive phylogenetic account of the origins of these genes, redefining each locus according to a positional criterion, through the integration of phyletic identity with comparative gene order information.

**Results:**

Each locus was individually specified by its surrounding gene order and associated with homologs showing the same position ('homoeologs') in other species, where available. Bayesian and maximum likelihood phylogenies were in general agreement on systematic relationships and confirmed several 'orthology sets' of genes retained since divergence from the common ancestor. Reconciliation analysis quantified the scale of duplication and gene loss, as well as identifying further apparent orthology sets, which lacked conservation of genomic position. These instances suggested substantial genomic restructuring or transposition. Other analyses identified clear instances of evolutionary rate changes post-duplication, the effects of concerted evolution within tandem gene arrays and gene conversion events between syntenic loci.

**Conclusion:**

Despite their importance to cell function and parasite development, the repertoires of AAT loci in trypanosomatid parasites are relatively fluid in both complement and gene dosage. Some loci are ubiquitous and, after an ancient origin through transposition, originated through descent from the ancestral trypanosomatid. However, reconciliation analysis demonstrated that unilateral expansions of gene number through tandem gene duplication, transposition of gene duplicates to otherwise well conserved genomic positions, and differential patterns of gene loss have produced largely customised and idiosyncratic AAT repertoires in all three species. Not least in *T. brucei*, which seems to have retained fewer ancestral loci and has acquired novel loci through a complex mix of tandem and transpositive duplication.

## Background

Amino acid transporter (AAT) proteins are crucial to the metabolism and physiology of trypanosomatid parasites [1]. Among these unicellular eukaryotes are *Trypanosoma brucei, Trypanosoma cruzi *and *Leishmania major*, which are causes of substantial human morbidity worldwide. These organisms have a digenetic life cycle, being transmitted into a vertebrate host from a haematophagous insect vector. The medical importance of these parasites prompted the recent completion of their genome sequences [2-4], which have provided an improved understanding of their genetic repertoire. Furthermore, greater appreciation of surface-expressed proteins regulating membrane transport may lead to new therapeutic targets or improved means of drug-delivery [5,6]. This study addressed the repertoire of AAT genes through the integration of phyletic and positional information, to identify the mechanisms by which new loci originated during the history of the Trypanosomatidae. Hence, the specification of loci across the family was explicitly phylogenetic, reflecting the histories of AAT genes, and spatial, using comparative gene order information to establish homoeology (i.e., orthologous genes found in conserved genomic positions).

The importance of AAT proteins to trypanosomatids as cell surface regulators of amino acid transport is manifold. Amino acids are used as primary energy sources during the insect stages, due to the relative oligotrophy of the vector midgut environment and the relative abundance of proline [7,8,1]. Arginine is also utilised as an energy reservoir when it is converted into phosphoarginine by arginine kinase in *Trypanosoma *spp. [9,10]. These substrates are so important that they are necessary for the culture of vector stages in *T. cruzi *and can ensure survival during starvation conditions [11,12]. Besides this, an intracellular pool of amino acids is permanently maintained by trypanosomatids for osmoregulation [13-15]. The various host environments of any trypanosomatid life cycle vary greatly in the osmotic stress they place on the parasite. Successful transition between host environments requires modulation of the intracellular osmolytes, which mostly comprise alanine, glycine, glutamate and ornithine [13]. For both energetic and osmotic reasons, the demands on AAT proteins vary as the parasite progresses through its life cycle; evidence suggests that ambient amino acid concentrations operate as cues for developmental differentiation and therefore, that AAT proteins act as physiological indicators during life stage transition [16-18]. Hence, efficient regulation of amino acid transport is not only vital for survival in particular life stages, it is also imperative for successful transition between stages.

Trypanosomatid genomes contain large numbers of AAT genes, often arranged in tandem gene arrays [2-4]. Transporters of specific amino acids and generic substrates are known [19,20], and expression of these genes can be linked to particular life stages. Distinct low- and high-affinity arginine transporters are expressed in both *T. cruzi *[21,22] and *L. donovani *[23]. In *T. cruzi *at least, expression is known to be stage-specific, with activity ceasing in bloodstream-form, non-replicating trypomastigotes [17]. Similarly, multiple proline transporters are known in *T. cruzi *[24] and *L. donovani *[25]. A proline-specific protein is active in the *L. donovani *promastigote (vector form), but silenced in the amastigote (vertebrate) form when proline ceases to be the primary energy source [25]. Hence, the diverse AAT repertoires of trypanosomatids appear to entail specialisation of individual loci to regulation of particular amino acids, and enable modulation of intracellular amino acid concentrations in response to changes in host environment. Most of these transporters have been biochemically characterised but not related to a specific locus in the genome sequence. While the capacity for differential expression of proteins with alternative substrate affinities is well established, corresponding sequence has only been obtained for a high-affinity arginine transporter in *L. donovani *[23], an amastigote-specific transporter in *L. amazonensis *[26] and a polyamine permease in *L. major *[27]. Other studies have detailed the sequence variation among AAT genes in *L. major *promastigotes [28] and *T. cruzi *[29], without individually characterising their products. From these it is clear that gene repertoire far exceeds what has been biochemically characterised, but these lists may not be complete and their specific classifications have not been harmonised.

This study applied a comparative approach to gene family evolution of AAT genes in trypanosomatids, with the principal objective of determining the origins of individual AAT loci and the mechanisms responsible for differences in repertoire between species. The three genome sequences were compared to identify all AAT loci and define each component as shared or distinct. Defined by their genomic position, one expects loci to be represented in multiple species, with all genes at that position being termed homoeologs. Exceptions to the pattern, for instance, failure to find orthologous sequences in the same location, or discovery of an unrelated sequence in a homoeologous position, are powerful indicators of evolutionary mechanisms. Hence, homoeology was inferred to classify all loci with unique *identities*, regardless of species. This protocol was compared with classifications already available for each individual species. Phylogenetic hypotheses of the entire gene family were estimated and patterns of duplication and loss were inferred through reconciliation analysis. Analysis of gene diversity was complemented with assessments of evolutionary rate changes in response to duplication and of the roles of concerted evolution and gene conversion in regulating sequence evolution.

## Results

The genome sequences of *T. brucei, T. cruzi *and *L. major *include 36 unique AAT loci, as specified by their genomic positions. These positions were inspected in each species to determine gene complement. Phylogenetic reconstruction of all AAT gene sequences using both Maximum Likelihood (ML) and Bayesian Inference (BI) methods was the basis for subsequent analyses of evolutionary rate changes and reconciliation analyses of gene duplication and loss. Finally, the effects of concerted evolution and gene conversion on AAT gene sequences, both within and between loci, were examined for their potential to introduce artefact into phylogenetic reconstruction and to regulate sequence diversity.

### AAT gene complement

The three trypanosomatid genomes display a generally conserved gene order, despite substantial karyotypic differences [3], allowing orthologous sequences to be connected by their genomic positions, specified by surrounding gene order. Even when the AAT gene was absent, the genomic position by which the locus was specified was typically conserved. The 36 AAT loci are listed in Table 1 and are hereafter referred to by their locus number and the initials of their host species; many of the loci comprised tandem-duplicated genes, producing a total number of 94 distinct gene sequences. The classification in Table 1 has been reconciled with previous classifications for each individual trypanosomatid species, for *T. brucei *[30], *T. cruzi *[29] and *L. donovani *[28]. Immediately, it is clear that these studies, which recorded distinct AAT sequences from tissue preparations or preliminary read libraries, found only a fraction of the total AAT gene diversity. However, TzPAT types 1 and 6 from *T. cruzi *found no direct match in the current *T. cruzi *genome sequence; these two sequences were previously identified from preliminary read data and confirmed using RT-PCR [29], and their absence from the completed genome sequence probably suggests sequence gaps, where there were insufficient reads to assemble a finish contig.

**Table 1:** Trypanosomatid amino acid transporter (AAT) loci: GeneDB identification, copy number and cross-references to other classifications.

AAT	Genome sequence:						Parallel classification:		
Locus	*T. brucei*		*L. major*		*T. cruzi*		*T. brucei*	*L. donovani*	*T. cruzi*

	ID	n	ID	n	ID	n*			

1	Tb927.4.3930	1	LmjF31.0320	4	Tc00.1047053508557.2.	1			TzPAT2
2	Tb927.4.3990	4	-	-	-	-			
3	Tb927.4.4730	1	-	-	-	-	AATP11		
4	Tb927.4.4820	6	-	-	-	-	AATP3, 7–10^#^		
5	Tb927.8.4700	6	-	-	Tc00.1047053506153.6.	2	AATP6		TzPAT4
6	Tb927.8.5450	1	-	-	-	-			
7	Tb927.8.7600	11	-	-	-	-	AATP1		
8	Tb927.8.7740	1	LmjF31.1790	3	-	-		AAP1LD	
9	Tb927.8.8220	5	-	-	-	-			
10	Tb927.8.8290	2	-	-	-	-	AATP5		
11	Tb09.211.1760	1	LmjF35.4410	1	Tc00.1047053509551.3.	1		AAP10LD	
12	Tb10.70.1170	1	-	-	Tc00.1047053509733.17.	1			
13	Tb10.70.0300	1	LmjF31.0870	2	Tc00.1047053510187.53.	1		AAP3LD	TzPAT9
14	Tb10.6k15.0450	1	LmjF36.4480	1	Tc00.1047053504147.6.	1			TzPAT11
15	Tb11.02.4520	1	LmjF11.0520	1	Tc00.1047053506773.1.	1			
16	Tb11.01.7500	2	LmjF32.2660	1	Tc00.1047053511545.70.	1			
17	Tb11.01.7590	2	-	-	Tc00.1047053511543.4.	1			TzPAT10
18	-	-	LmjF02.0440	1	-	-			
19	-	-	LmjF07.1160	1	-	-			
20	-	-	LmjF10.0710	2	-	-			
21	-	-	LmjF14.0320	1	Tc00.1047053504213.12.	1			TzPAT12
22	-	-	LmjF22.0230	1	-	-			
23	-	-	LmjF27.0670	2	-	-			
24	-	-	LmjF27.1580	1	-	-		AAP15LD	
25	-	-	LmjF31.0570	2	-	-		AAP2LD	
26	-	-	LmjF33.1420	1	Tc00.1047053511837.9.	1		AAP13LD	
27	-	-	LmjF35.5350	2	Tc00.1047053510431.2.	1		AAP8LD, AAP11LD^†^	
28	-	-	LmjF36.0420	1	-	-			
29	-	-	LmjF36.6830	1	-	-			
30	-	-	-	-	Tc00.1047053510507.40.	1			TzPAT8
31	-	-	-	-	Tc00.1047053509167.40.	1			
32	-	-	-	-	Tc00.1047053504069.120.	1			
33	-	-	-	-	Tc00.1047053506227.10.	1			TzPAT5
34	-	-	-	-	Tc00.1047053507659.30.	1			TzPAT3
35	-	-	-	-	Tc00.1047053511649.100.	1			TzPAT7
36	-	-	-	-	Tc00.1047053504229.110.	1			

* Copy number in *T. cruzi *is not well determined; hence many loci with a single recorded gene copy may comprise more.

# AAT4 specifies a tandem gene array of six copies; each copy was given a different AATP number in the classification of Hasne 2002 but all derive from the same genomic position.

† AAT27 specifies a tandem pair; AAP8LD and AAP11LD refer to the first and second copies respectively in the classification of Akerman et al. 2004.

**Figure 1:**
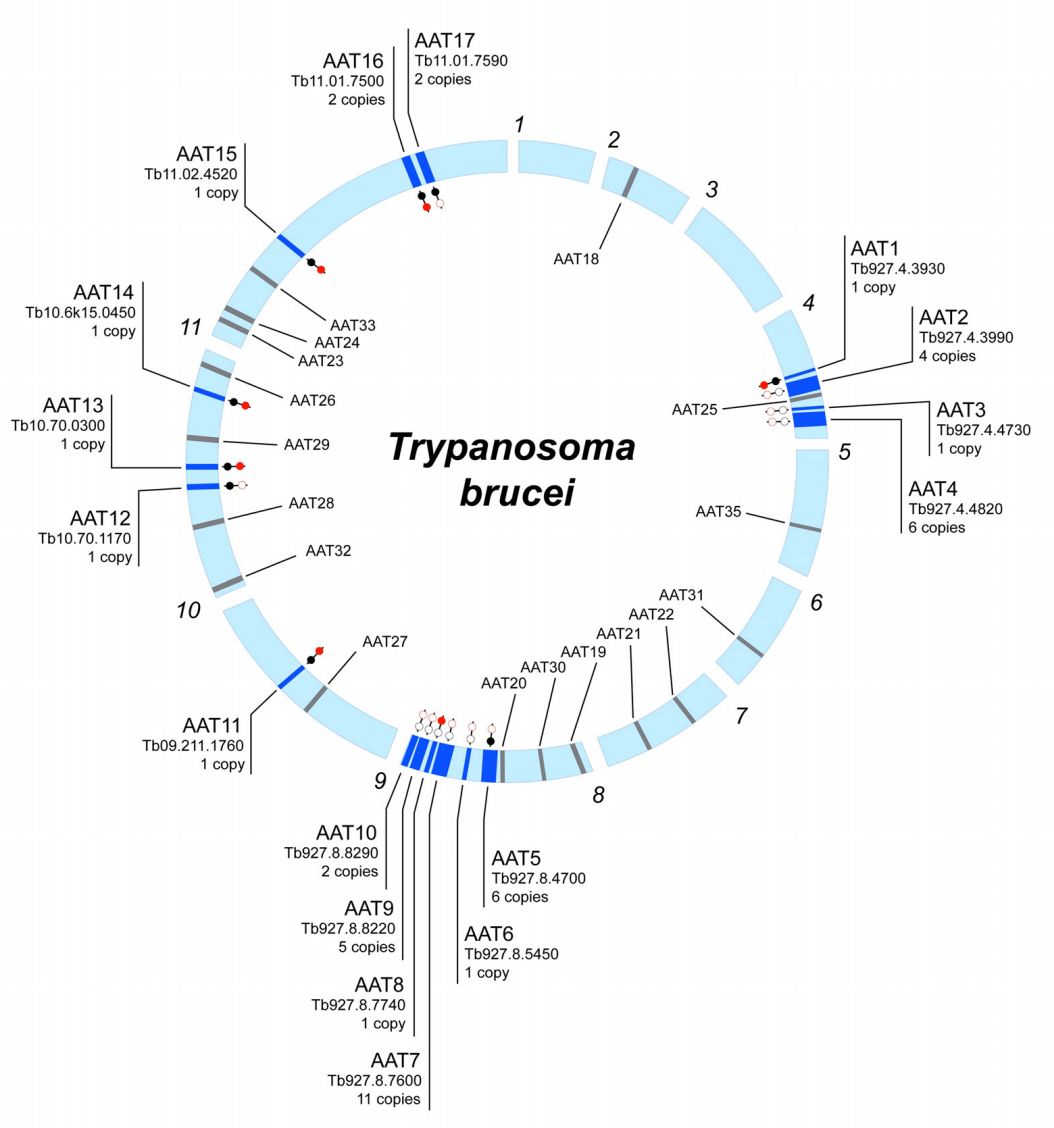
*Trypanosoma brucei *AAT loci. 11 Chromosomes are arranged circularly and labelled by number in clockwise fashion. Dark shaded bars across chromosomes represent AAT loci and are labelled with locus number, GeneDB identifier and copy number (also reflected in the band width). Grey shaded bars represent the genomic positions of AAT loci found in *L. major *or *T. cruzi*, but absent in *T. brucei*, and are labelled inside the circle. The status of each AAT locus in *L. major *and *T. cruzi *is represented by red and black circles respectively; shaded circles indicate the presence of a homoeologous gene, open circles indicate the absence of any AAT gene, but with typically conserved synteny around the location.

**Figure 2:**
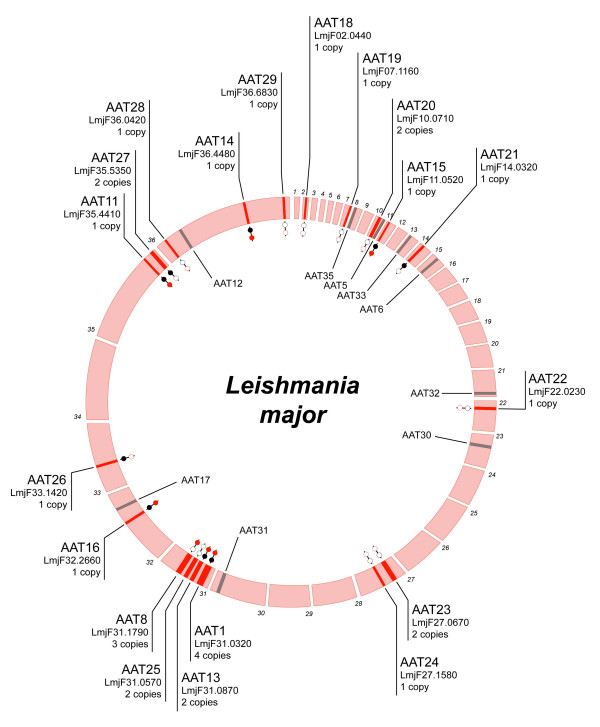
*Leishmania major *AAT loci. 36 Chromosomes are arranged circularly and labelled by number in clockwise fashion. Labelling is applied as in Figure 1; grey bars inside the circle represent the homologous positions of AAT loci present in *Trypanosoma *spp. but absent in *L. major*; the presence of homoeologous gene copies in *T. brucei *and *T. cruzi *is identified by red and black dots respectively.

Comparison of AAT gene complements showed that 6 loci are present in all species (AAT1, 11, 13–16), although their copy number varied, for example, AAT1 included 4 copies in *L. major *but was single-copy in *Trypanosoma *spp. Other loci were shared by two of three species and indicated gene losses. For instance, 3 loci were shared by *L. major *and *T. cruzi*, but not *T. brucei *(AAT 21, 26, 27). Similarly, AAT8 was not found in *T. cruzi*. A further 3 loci were found in *Trypanosoma *spp. but not *L. major *(AAT5, 12, 17), suggesting either loss or an origin after the separation of genera. All loci are shown in Figures 1 and 2 with the conserved positions of absent AAT genes (shaded grey). For both *T. brucei *and *L. major*, the number of vacant positions (and, conversely, of species-specific loci) in these figures indicates that each species has a largely customised AAT gene repertoire, with 7/17 *T. brucei *loci, 9/19 *L. major *loci and 7/19 *T. cruzi *loci being species-specific respectively.

### AAT gene family phylogeny

**Figure 3:**
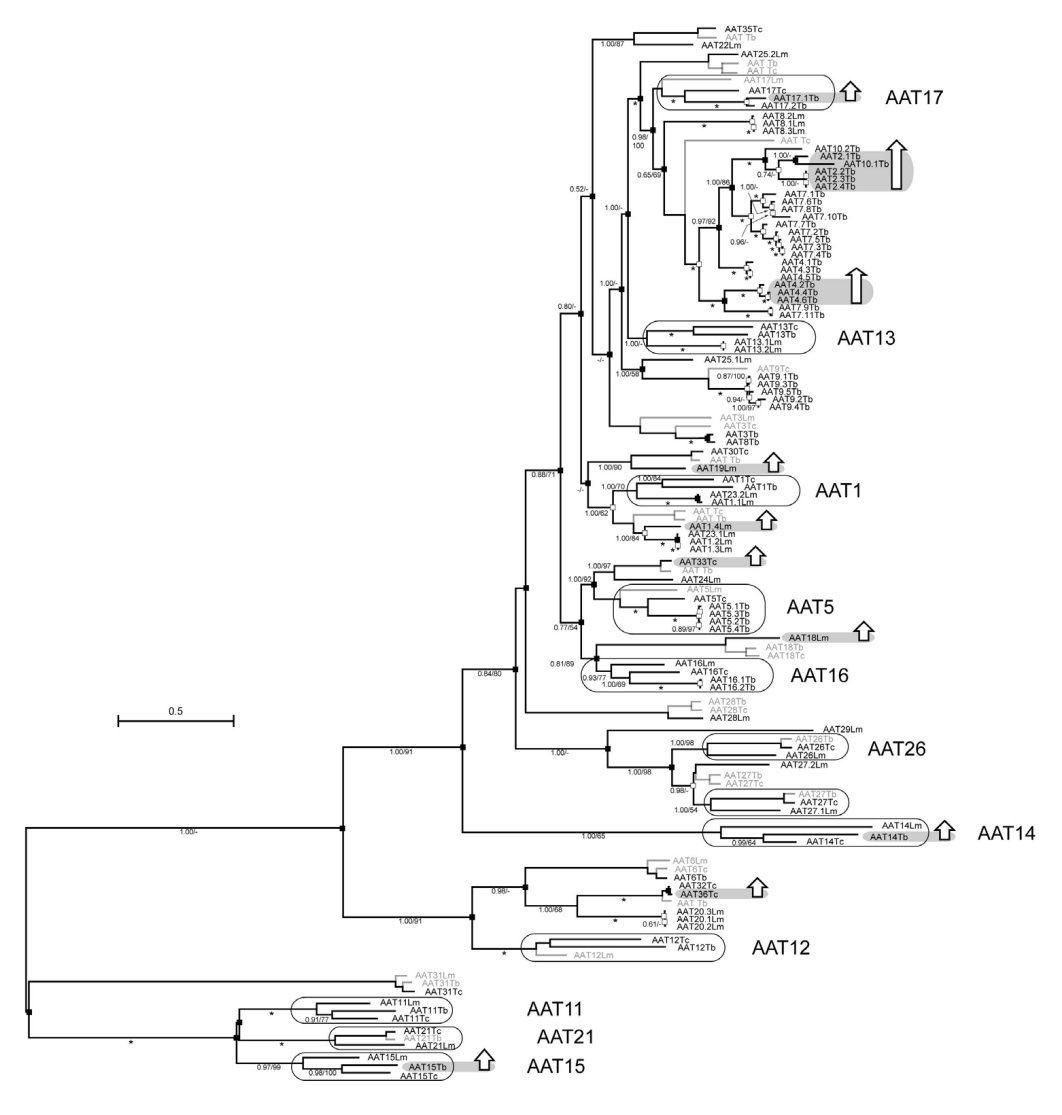
Maximum likelihood molecular phylogeny of trypanosomatid AAT gene sequences. Values attending branches represent Bayesian posterior probabilities, followed by non-parametric bootstrap proportions, out of 100 replicates. Asterisks * denote values of 1.00/100 for a given node. Dashes – represent missing values or bootstrap proportions lower than 50. Duplications inferred by reconciliation analysis are indicated by shaded squares (transpositive duplications) and open squares (tandem duplications). Losses inferred by reconciliation analysis are indicated by 'ghost' branches, shaded faintly. Clusters of homoeologs from different species ('orthology sets') are bordered and labelled with their locus identifier in large type. Those sequences showing significantly excessive evolutionary change relative to an outgroup, (as determined by relative rate test), are shaded alongside a vertical open arrow.

Figure 3 shows a ML phylogenetic tree estimated for 93 AAT gene sequences, with substitution rates specified by a GTR+I+Γ model. As shown by the high Bayesian posterior probabilities and bootstrap values at most basal and apical nodes, ML and BI methods produced mutually consistent estimates. Both the basal nodes (between AAT14 and AAT15) and crown clades in the tree were robust. Many of these clades corresponded to 'orthology sets' of gene sequences from two or all three species; for example, homoeologs for AAT11, 13, 15 and 16, which were present in all species, clustered robustly. Hence, with a few exceptions (see below), these clades coincided with a shared genomic position. Several nodes at the base of larger clades lacked robustness and are shown without support measures, or with posterior probabilities only. These nodes occur between AAT16 and AAT17 and correspond to the relationships between the major crown clades. In the Bayesian tree (not shown) these nodes were collapsed, and were the only difference between the BI and ML phylograms. Application of a covarion model to Bayesian inference made no difference to the tree topology.

Most genes defined as homoeologs based on genomic position fulfilled expectation by clustering together in the phylogeny. However, tandem gene duplicates routinely clustered together, suggesting that the arrays were species-specific and recently evolved. Furthermore, these arrays often did not have homoeologs in other species. For example, AAT2, 4, 7 and 10 in *T. brucei *contained many tandem duplicates; this substantial expansion that was most closely related to AAT8Lm and AAT17. The genomic locations of these loci, on chromosomes 4 and 8 in *T. brucei*, were without clear correspondence in the *L. major *or *T. cruzi *genome sequences, as shown in Figures 1 and 2, indicating that these loci were novel, or at least rearranged. In *L. major*, AAT1, 8 and 20 included tandem duplicates and these clustered together, despite the existence of homoeologs in the first two cases.

The clustering of homoeologs was expected, given that the affinity between related gene sequences should reflect the common identity imparted by shared genomic position. However, Figure 3 shows several close and robust relationships between *L. major *and *T. cruzi *loci, where the genomic position in *T. cruzi *was conserved *in L. major *without any trace of an AAT gene (Figure 2). They include AAT19lm/AAT30Tc, AAT20Lm/AAT32Tc, AAT22Lm/AAT35Tc and AAT24Lm/AAT33Tc. In these examples, sequence identity suggests an affinity, but positional identity does not. In other situations, genes sharing positional identity are unrelated in gene sequence. AAT23Lm is a tandem pair; however, the two copies are not closest relatives and display a more complex relationship with AAT1. AAT25Lm is also a tandem pair but its two copies are unrelated, and rather than clustering with homoeologs in *L. major*, AAT8Tb is almost identical to AAT3Tb.

### Reconciliation analysis of duplication and loss

**Figure 5:**
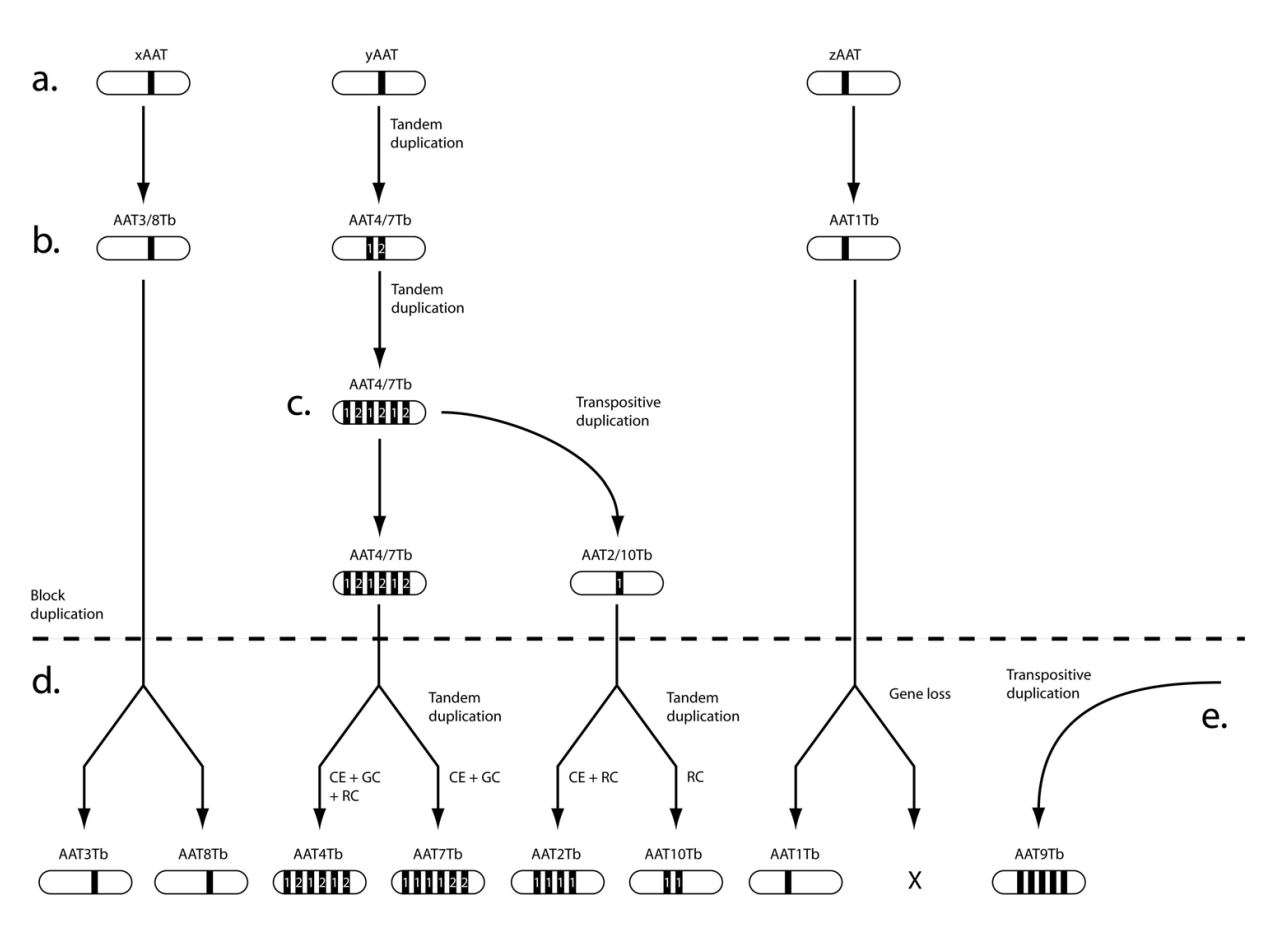
Hypothesis for the origin of AAT loci on chromosomes 4 and 8 in *T. brucei*. a. Three gene lineages were present on the ancestral chromosome; these were ancestral to the AAT3/AAT8 loci, the AAT4/AAT7 loci and the AAT1 locus respectively. b. Tandem duplication created a dimorphic locus, ancestral to the contemporary AAT4 and AAT7 loci. c. A transpositive duplication of one sequence type from AAT4/7 produced the monomorphic AAT2/10 locus on the same chromosome. d. A block duplication of the chromosome (denoted by dashed line) resulted in two paralogons (now attached to chromosome 4 and 8). This duplicated the ancestral gene lineages, creating the contemporary loci. Among AAT4 and AAT7 other events affected gene number and sequence: CE – concerted evolution, GC – gene conversion, RC – evolutionary rate change. AAT1 has no paralog on chromosome 8, and so must have been lost. e. AAT9 must have been transposed later since it has no paralog on chromosome 4, and is unrelated to the other loci.

Incongruence between the gene and species trees was reconciled to produce an exhaustive list of duplications and losses; these are mapped on to the gene tree in Figure 3. Duplications were classified as either 'transpositive' or 'tandem', depending on whether the duplicate moved to a new location or not. Basal nodes reflect ancient, transpositive duplications, although these also occurred within crown clades. For example, the close relationships between AAT32Tc andAAT36Tc, as well as AAT3Tb and AAT8Tb, were interpreted as transposed duplications. The substantial expansions of certain clades in *T. brucei *and *L. major *were interpreted as tandem duplications, with occasional transposition. AAT1Lm expanded to 4 copies through tandem duplication; the first copy was retained in all three species. Transposition of the first and second copies was required to account for AAT23Lm, which is a tandem pair that clustered within the AAT1 clade (as described above). Likewise, the AAT2/4/7/10 clade originated through tandem duplication with periodic transposition, creating several loci with multiple gene copies (see Figure 5 below).

Since all species should have orthologs for a given lineage if it was present in their common ancestor, losses were invoked where orthologs were not evident. Where orthologs were present, even if non-homoeologous, neither duplication nor loss was required; such loci originate through descent from the ancestor. The presence of homoeologs in two species was explained by loss in the third species; AAT5Lm, AAT12Lm, AAT17Lm, AAT21Tb, AAT26Tb and AAT27Tb were all deleted (or transposed) after separation of the three species. Losses were also invoked for those clades where sequence affinity suggested orthology, but positional identities were distinct, for instance AAT22Lm/AAT35Tc and AAT19Lm/AAT30Tc. As stated previously, these mostly concerned closely related sequences from *L. major *and *T. cruzi*, and so the would-be ortholog in *T. brucei *was lost.

The overall picture derived from reconciliation analysis is summarised by the total numbers of duplications and losses experienced by each species. *T. cruzi *and *L. major *experienced few transpositive (1 and 3 respectively) and tandem (0 and 7 respectively) duplications, while *T. brucei *experienced many more (5 and 26 respectively). However, these figures depend on the precise assembly of repetitive genome sequences, and are probably not accurate (see discussion). The number of loss events however, is determined by presumed orthologs that are absent, and hence, directly reflects the relative AAT complement in each species. *T. brucei *had the more losses (13) than either *T. cruzi *(9) or *L. major *(6). Considering both the obvious orthology sets and those inferred through reconciliation analysis, *T. brucei *lacks representatives in these clades more often that the other two species. This suggests that *T. brucei *has experienced greater losses of its inherited AAT complement.

### Evolutionary rate changes

Relative rates tests were applied to every terminal branch in the gene tree, after separating the tree into 26 different subclades. Additional file 1 (and also Figure 3) describes those tests that recorded a significant difference in evolutionary change between two lineages. Several lineages showing significantly greater evolutionary change than their sister lineages or clades derived from duplication events. The clade comprising AAT2Tb and AAT10.1Tb had an accelerated substitution rate relative to AAT10.2Tb (transpositive duplication). The transpositive duplications affecting AAT32/AAT36 and AAT16/AAT18 both coincide with significant substitution rate accelerations by AAT36Tc and AAT18Lm respectively. The clade including AAT4.2 and two other copies from that array shows a highly significant elevation in evolutionary rate, relative to the sequences of AAT7Tb and AAT10.2Tb. Finally, AAT17.1Tb showed a greater amount of evolutionary change than its tandem duplicate AAT17.2Tb. Other cases involve genes originating through descent, rather than duplication. Among AAT14 orthologs, the *T. brucei *copy has experienced a significantly higher substitution rate than the *T. cruzi *gene. While among AAT15 orthologs, the *T. brucei *gene shows an excess of substitutions relative to the *L. major *gene, but not to the *T. cruzi *copy. Similarly, AAT19Lm has changed significantly more than its putative ortholog AAT30Tc, and AAT33Tc showed an accelerated substitutional rate relative to its putative ortholog AAT24Lm.

### Effect of concerted evolution

**Figure 4:**
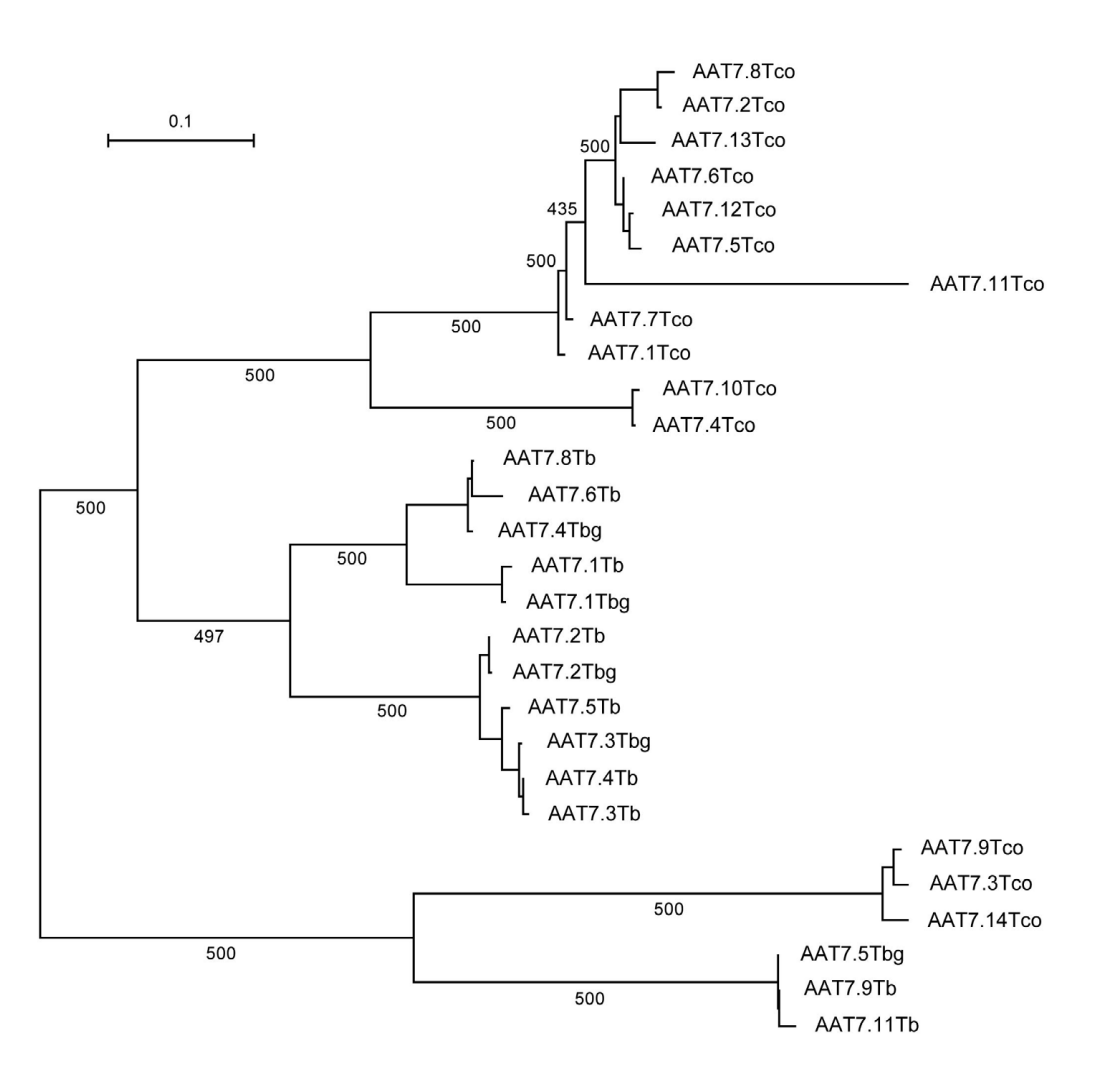
Maximum likelihood molecular phylogeny of AAT7 gene duplicates from *T. brucei*, showing evidence for concerted evolution within species. Sequences from *T. brucei *(Tb), *T. brucei gambiense *(Tbg) and *T. congolense *(Tco) were analysed. Values attending branches indicate non-parametric bootstrap proportions, out of 500 replicates. The phylogeny was unrooted and branch length varies according to the scale shown.

Concerted evolution was detected using a cladistic criterion whereby duplicate sequences were combined in a phylogenetic tree with homoeologous sequences from closely related species; concerted evolution was inferred where conspecific sequences clustered together to the exclusion of orthologs elsewhere, measured using SH tests. Of those loci with >2 gene duplicates, AAT4Tb, AAT9Tb and AAT8Lm could not be tested because no homoeologous loci existed in the appropriate species. As Table 2 shows, AAT2Tb and AAT5Tb gave unambiguous evidence for concert evolution of gene duplicates; where homoeologs from the various species were constrained to show orthologous relationships, this topology was significantly less likely than the optimal tree topology, unlike the topology constrained to show sequences clustering by species. In the cases of AAT7Tb and AAT1Lm, there were significant differences between optimal tree topologies and both hypotheses, suggesting that neither pure orthologous nor pure concerted evolution scenarios were sufficient explanations of sequence relationships. Figure 4 shows the optimal ML tree topology for AAT7Tb and explains why a tree topology purely reflecting concerted evolution was inadequate. In fact, AAT7Tb comprises two lineages: AAT7.9Tb and AAT7.11Tb comprised one lineage and have remained distinct from the remaining 7 duplicates in the array. Duplicates within each of these 'sub-arrays' evolved in concert, since *T. brucei/T. b. gambiense *copies clustered apart from *T. congolense *sequences. But the presence of the two sub-arrays ensured that any tree constrained to make all sequences monophyletic was sub-optimal.

**Table 2:** Analysis of concerted evolution among AAT tandem gene arrays in *T. brucei *and *L. major*.

AAT	Average sequence divergence:		Log Likelihood:						
Locus	Ks	Ka	Optimal	Orthologous			Concerted		

					Δ-lnL	P		Δ-lnL	p

AAT1Lm	0.7439	0.3554	-9270.9	-9803.67	-532.8	**0.0001**	-12761.51	-3490.6	**0.0001**
AAT2Tb	0.3427	0.1226	-6465.23	-6892.64	-427.41	**0.0001**	-6465.23	0	0.749
AAT5Tb	0.0127	0.0062	-4798.5	-6752.3	-1953.8	**0.0001**	-4872.6	-74.1	0.11
AAT7Tb	0.7609	0.1607	-5761	-6677.5	-916.5	**0.0001**	-6844.1	-1083.1	**0.0001**

### Effect of gene conversion

The GENECONV and SISCAN programs were used to detect unexpected sequence similarities within multiple alignments of gene duplicates, which were checked by eye and interpreted as evidence for gene conversion. Table 3 describes 7 such events, affecting 3 different tandem gene arrays in *T. brucei*. No further putative events were confirmed in other tandem arrays, or between unlinked AAT loci. AAT4Tb is a tandem gene array of six copies, which comprise two distinct sequence types that alternate along the array. For a 25 bp window, AAT4.2Tb was identical to the other sequence type (i.e., AAT4.1, 4.3 or 4.5), while almost identical to AAT4.4 and 4.6 for the remainder of its length. It was therefore obvious that AAT4.2Tb had been partially converted by a copy of the second sequence type. Both GENECONV and SISCAN confirmed that this was a significant anomaly and indicative of gene conversion. Similarly, AAT9Tb is a tandem gene array of 5 copies, where the first and third duplicates clustered together, apart from the remaining genes. However, for a 151bp window towards the C-terminus AAT9.5Tb was identical to the third duplicate, causing a significant phylogenetic inconsistency that was interpreted as partial gene conversion of the 3' end of AAT9.5Tb by AAT9.3Tb. Finally, there were several phylogenetic anomalies observed among the 11 gene duplicates of AAT7Tb; frequent exchange of sequence motifs was consistent with the high genetic variability among these genes (see Table 2).

**Table 3:** Gene conversion events between AAT gene duplicates.

Identifier	Recombinant region:			P-value		Donor	Recipient
	Length	Start	Finish	GENECONV	SISCAN		

AAT4Tb	25	511	536	6.07e-06	5.78e-05	1, 3 or 5	2
AAT7Tb	48	1	49	1.02e-03	3.42e-05	8	5
AAT7Tb	205	1	206	1.50e-17	2.08e-06	3	1
AAT7Tb	81	548	629	6.95e-03	2.56e-05	4	1
AAT7Tb	19	1042	1061	2.59e-03	3.83e-03	9	1
AAT7Tb	151	1086	1237	1.03e-05	2.44e-03	6	7
AAT9Tb	327	739	1066	7.89e-17	1.25e-18	3	5

## Discussion

The AAT loci in *T. brucei, L. major *and *T. cruzi *were defined by their phylogenetic relationships and shared genomic positions. Previous classifications of AAT loci from individual species were harmonised and it was shown that none entirely encompassed the diversity evident from genome sequences. Reconciliation analysis of the gene family phylogeny identified numerous gene losses and various transpositive and tandem duplications. Some among these duplications were associated with significant changes in evolutionary rate, while others were affected by concerted evolution and gene conversion. These various observations accounted for the quite distinct AAT complements in each organism, with only 6 out of 36 loci being present in all three species. Accordingly, while descent from a common ancestor was the most parsimonious origin for these and some other loci, most other loci originated through lineage-specific evolutionary innovations.

### Origin by descent

Certain AAT loci in each species were inherited from the common ancestor of all three. These genes formed orthologous clusters in the gene tree and displayed conserved genomic positions in all three species, or sometimes just in two. Many of these loci, such as AAT11, 12, 15 and 21, are found in basal positions and could be viewed as essential genes, loss of which results in negative selection. Their position reflects a recognised difference in protein family; they belong to the amino acid polyamine-choline superfamily, while the majority of loci in Figure 3 belong to the amino acid transporters-1 superfamily. Hence, the functional differences between amino acid transporters and permeases, (i.e., the transport of molecules with single and multiple amino groups respectively), may account for the observed distinction in evolutionary dynamics. Amino acid permeases have been both lost and duplicated less often. Among other more 'stable' loci, AAT13, 16 and 17 are amino acid transporters that have originated through descent, without frequent loss or duplication.

There may be other cases of origin by descent inferred by reconciliation analysis. For clades such as AAT19lm/AAT30Tc, AAT20Lm/AAT32Tc, AAT22Lm/AAT35Tc and AAT24Lm/AAT33Tc the high sequence identity between loci at different genomic positions indicated orthology, but apparently no homoeology. They may have originated through transpositive duplication in one or both species, coupled with deletion of the original locus, or through a radical rearrangement of surrounding genes, such that conserved synteny was lost. Notably, each of these cases involved *L. major *and *T. cruzi *and consequently, the number of inferred losses in *T. brucei *was increased commensurately.

Other cases of origin by descent also involved duplication events, such as AAT1 where an orthologous set is sister clade to a number of gene duplicates in *L. major*, and AAT5, where the locus is arrayed in *T. brucei*, but a singleton in *T. cruzi*. However, in the context of employing amino acid transporter proteins as a means for importing trypanocidal drugs into the parasites, these loci that show less flexibility through time, less propensity for duplication, functional differentiation or loss, could be superior targets since they are less likely to show functional redundancy or sequence variation in the form of tandem duplicates. Their long-standing orthology also suggests that they have also proven less tolerant of disruption in the past.

### Origin by transpositive duplication

Reconciliation of gene and species trees explained the disparity between gene and species numbers through duplication events. The oldest nodes of the gene tree are depicted as a series of transposition events, which one can interpret as occurring between genomic locations, giving rise to the contemporary crown clades. However, these events are placed at the time of an ancestor of all trypanosomatids, and the chromosomes in each extant species probably did not exist in this ancestor. Expansion of the gene family coincided with the evolution of contemporary trypanosomatid genome structures. One can but speculate on these evolutionary changes, but they must have involved chromosomal duplications and fusions, changes in ploidy and gene capture (xenology). *T. brucei *has certainly experienced substantial karyotypic evolution through chromosomal fusion [31] and this will have affected its AAT repertoire. Yet the basal nodes in Figure 3 remain in the very distant past, and it is here that implicit assumptions of this study, that phylogeny is dichotomous and genomic position is conserved, will be most precarious.

Transpositive duplication is also inferred in the crown clades, where nearest relatives are found in dissimilar genomic positions. AAT32Tc/AAT36Tc are almost identical, yet AAT32Lm and AAT20Lm appear to be orthologs, while AAT36Lm is found in a subtelomeric region amongst arrays of other high copy gene families. AAT23Lm is a tandem pair that resulted from the transposition of the first two AAT1 gene copies to a new location; it is likely that only two AAT1 gene copies existed at that time, tandem duplication has caused subsequent expansion. In some cases, transpositive duplication coincided with acceleration of evolutionary rate (or deceleration in the sibling lineage), which is indicative of functional differentiation [32], for example, AAT36Tc (transposed to a subtelomeric region) has an accelerated substitution rate relative to AAT32Tc; AAT2Tb (originated as part of a block duplication) also has an accelerated evolutionary rate relative to other loci on chromosomes 4 and 8. The combination of transpositive and tandem duplication is demonstrated well by the loci on chromosomes 4 and 8 in *T. brucei*, described in Figure 5. This considerable expansion spans four locations and originated through an initial tandem duplication to create a dimorphic array (which is now either AAT4Tb or AAT7Tb). Subsequently, a transposition event from one copy within the dimorphic array established the AAT10/AAT2 clade. The current situation, with AAT2 and AAT4 on chromosome 4, AAT7 and AAT10 on chromosome 8, and AAT4/7 and AAT2/10 being paralogous pairs, is best explained through a block duplication event creating two duplicons, after the initial tandem and transpositive duplications. This being so, the phylogenetic position of AAT3Tb and AAT8Tb, which should also be a paralogous pair affected by the block duplication (on the basis of genomic position), is puzzling (see below). Finally, the origin of AAT9Tb, found on chromosome 8 but unrelated to any of the afore-mentioned paralogous pairs, must also be transpositive; these genes are also only found on one of the putative duplicons (i.e., chromosome 8).

### Origin by tandem duplication

Tandem gene arrays are among the most challenging to resolve correctly using current genome sequencing technology, since repetitive sequence reads tend to collapse into a single contig when no variation exists to distinguish them. Hence, it is difficult to determine the presence and length of tandem arrays; so where variation exists it is likely that complete genome sequences will contain all distinctive gene duplicates, but not the correct number. In general, the patterns of tandem duplication are idiosyncratic. Some comparisons are reliable, for example, AAT1Lm shows tandem duplication, while AAT1Tb does not. However, the apparent abundance in *T. brucei *may say more about the ability to detect tandem duplicates in *L. major *and (especially) *T. cruzi*, than a real dynamic difference. Confirmation of tandem gene arrays is possible because most tandem duplicates contain sequence variation, often arranged as distinct sequence types. Indeed, distinct sequence types characterised by previous studies can now be seen to originate from a single array; AATP3, 7–10 [30] all derive from AAT4Tb. Similarly, AAP8LD and AAP11LD are distinct genes in *L. donovani *[28], and these correspond to the tandem pair in *L. major *at AAT27.

Clearly, it is possible for duplicates to become distinct and maintain their integrity within the array, for instance, the scenario described in Figure 5 suggests that the two distinct sequence types seen within both AAT4Tb and AAT7Tb are long-standing. Such discontinuous variation indicates that tandem duplication may be followed by adaptive divergence, in the manner of a duplication-divergence-complementation model [32-34], to facilitate the expression of AATs in specific life stages, or for particular functions. It is notable that this is achieved within tandem gene arrays since their particular structural features indicate that variation would ordinarily be removed by concerted evolution (see below). However, functional differentiation may be promoted by the rapid changes in evolutionary rate that occasionally coincided with tandem duplication; for instance, AAT17.1Tb had an accelerated substitution rate relative to AAT17.2Tb, while AAT1.4Lm compared similarly to other gene copies within AAT1Lm. In contrast to such diversifying processes, the phylogenetic relationships of tandem duplicates from closely related *Trypanosoma *spp., and conflicting phylogenetic signals from alignments of tandem duplicates, indicated that both concerted evolution and gene conversion affect tandem duplicates in *T. brucei*. Partial allelic gene conversion between tandem duplicates was observed within three different arrays (see Table 3), although ectopic gene conversion between loci was never observed. This is consistent with gene conversion occurring proportionally according to physical proximity and sequence identity [35-38]. In the short term such small exchanges would work to diversify sequences, and may contribute to the generally high level of variation. However, a high rate of genetic exchange would eventually homogenise, and may then contribute to concerted evolution of gene duplicates.

For its part, concerted evolution was inferred for AAT2Tb, AAT5Tb and, to some extent, AAT7Tb, after significant clustering of *T. brucei *tandem duplicates, relative to homoeologs in *T. congolense*, was observed. The alternative explanation is that the heterospecific arrays are convergent, but the presence of a tandem array at these locations in the ancestor of the African trypanosomes is more parsimonious. AAT7Tb is an interesting case, since it suggests how tandem gene duplicates can both evolve in concert within a species, although still contain variation (as shown in Table 2). Concerted evolution will homogenise tandem gene duplicates through unequal crossing-over between sister chromatids or chromosomes, or through allelic gene conversion. Both mechanisms rely on mis-alignment of repetitive structures [39]. AAT7Tb contains two distinct sequence types that, as Figure 5 suggests, have retained their distinct identities despite proximity and identity with each other. The two forms are dissimilar enough to preclude misalignment with each other, but duplicates of each are not variable enough to prevent concerted evolution among each form. Hence, copies of each evolve in concert, but these collectively retain orthology with homoeologs in *T. congolense *(and therefore, failing both 'concerted evolution' and 'orthology' SH tests in Table 2).

### Parasite evolution and AAT repertoire

A combination of gene loss, transposition and tandem duplication has generated quite distinct AAT repertoires in trypanosomatids; also, gene sequences have often been affected in a species-specific manner by conservative and diversifying evolutionary pressures. Hence, the AAT repertoire is relatively labile, raising the question of how origination of novel loci and loss of established genes are regulated. At a proximate level, processes causing duplicated genes to be maintained at a new location or within an array have already been discussed: neo-functionalisation through accelerated divergence from the ancestral gene will ensure that subsequent loss is deleterious, while divergence, perhaps associated with gene conversion, will reduce the chances of deletion through unequal crossing-over within a tandem array. Those AAT loci characterised thus far suggest that the ultimate causation for changing AAT repertoires will derive from the specific needs of life stages and the particular properties of substrates. For example, AAT25Lm is a tandem pair, showing 84% identity. The two genes are not represented in *Trypanosoma *spp., although the surrounding gene order is very well conserved. The first of these genes was previously characterised in *L. donovani *(AAP2LD, [28]), and expressed in promastigote-stage cells (i.e., the vertebrate-infective stage). Conversely, the second gene was characterised in *L. amazonensis*, and shown to be expressed in the amastigote (i.e., the vertebrate, intracellular stage) [26]. Hence, in this instance, the duplication event may have been maintained due to sub-functionalisation of the tandem duplicates, resulting in their stage-specificity. Although these genes show a relatively high identity, they are not placed together in Figure 3, and this suggests that they are not tandem duplicates, but have come together at AAT25Lm through transposition or ectopic gene conversion. However, neither gene at AAT25Lm was placed robustly in the phylogeny.

Trypanosomatids have different amino acid requirements at each point in the life cycle. This occurs for both energetic and osmotic functions and relates to the very different chemical environments of the vector and vertebrate hosts. In a more general sense a suite of transporters are required for different substrates; AAT11 (corresponding to AAP10LD, [28]) and AAT21 (corresponding to TzPAT12, [29]) are positioned basally in Figure 3 and have been previously characterised as transporters of polyamines, rather than amino acids. These loci, and other probable polyamine transporters positioned basally such as AAT15 and AAT31, can be aligned with other amino acid transporters. They provide an obvious demonstration of how the gene family has diversified to transport distinct substrates. At a subtler level, proliferation of amino acid transporters may have facilitated specialisation to certain amino acids, according to need [24,17]. High affinity transporters of proline and arginine have been characterised and may have evolved since these particular substrates are of energetic importance [23,25]. AAT13 corresponds to a known high-affinity arginine transporter [23]. Low affinity transporters also regulate proline and arginine [25,22] but are able to transport many other amino acids, such as methionine [19] and glutamate [20]; AAT17 may correspond to a proline transporter [29]. To summarise, experimental evidence suggests that the precise functions of AAT loci relate to both substrate and to life stage. A combination of transposition and tandem duplication have provided the raw material for expansion of the gene family, which ultimately allows the parasite to manage its total amino acid content, and specific amino acid concentrations where these perform additional functions, as in the cases of proline and arginine.

## Conclusion

The origins of amino acid transporter loci in trypanosomatid parasites are complex, with evidence for both changes to gene complement and to gene sequences. Given that AAT loci have identities that transcend and predate the histories of specific genomes, definition of AAT loci according to their genomic positions and phylogenetic relationships, (i.e, by homoeology), was a successful strategy. Reconciliation of the gene tree with the species relationships indicated that gene complement has evolved through both transposition and tandem duplication. Often these duplicative origins were accompanied by conversion or rapid divergence of gene sequences, and experimental evidence elsewhere suggested that the novelty created by duplication is maintained to satisfy the requirements of particular life stages or for precise regulation of specific amino acids. While the repertoire of polyamine transporters was largely conserved in all three species (though not entirely without gene losses), the amino acid transporter complement showed substantial interspecific variation. This was exemplified in *T. brucei *where deletions of ancestral loci, combined with the derivation of many new genes through a chromosomal, block duplication, has generated a different AAT repertoire to either *L. major *or *T. cruzi*. Therefore, despite their importance to cell development and function, or perhaps because of this, and despite the general conservation of gene order among these three trypanosomatids, AAT loci have proven evolutionarily labile and prone to repeated innovation.

## Methods

### Sequence retrieval and locus specification

AAT gene sequences were obtained from GeneDB [40]. The genome sequences for *T. brucei, T. cruzi *and *L. major *were searched for annotated amino acid transporters. To check for unannotated sequences that could be aligned, AAT genes were BLASTed against all coding sequences for each species. Gene loci were then specified by genomic position. Starting with *T. brucei*, each AAT locus was defined by the surrounding gene order. Where multiple genes were arrayed, the whole array was considered a single locus. This resulted in 17 unique loci in *T. brucei *(labelled AAT1-17). The *L. major *genome sequence was then inspected; where a gene displayed conserved synteny with a *T. brucei *locus, it was assigned the same identity, and the corresponding genes were considered homoeologous. This resulted in 19 loci, 7 of which were present with conserved synteny in *T. brucei*, and a further 12 loci with unique identities (AAT18-29). Finally, AAT loci in *T. cruzi *were identified; the presence of AAT1-29 was checked by using the surrounding gene order in *T. brucei *and/or *L. major *to search the *T. cruzi *genome sequence. Where an AAT gene was found in a corresponding position, the locus was again given the same specification. This resulted in 12 homoeologous AAT loci. After discounting these from all available AAT genes in the *T. cruzi *genome sequence, this left an additional 7 genes in genomic positions that were not represented in either *T. brucei *or *L. major *(AAT30-36). For two of these genes (AAT34 and 36), their position on small contigs suggested that they were distinct within *T. cruzi*, but meant that there was insufficient positional information to assess homoeology. Hence, a total of 36 loci showing unique positional identities were specified across three species.

### Sequence alignment

All gene sequences, including gene duplicates within tandem gene arrays, were aligned using ClustalX [41]. Sequences were labelled by their locus and species, for example, AAT5Tc describes AAT5 in *T. cruzi*. Tandemly-arrayed genes were given sequential labels, for example, in *T. brucei *where AAT5 is present with four arrayed copies, these are labelled AAT5.1Tb to AAT5.4Tb. Sequences were aligned after translation to maximise the observed conservation and then end-trimmed to remove the amino- and carboxy-termini that could not generally be aligned. The remaining conserved domains comprised 2199 bp, with considerable gaps inserted due to the difference in length between AAT14Lm (2349 bp) and most other loci (~1400 bp). All subsequent analyses were carried out on the back-translated alignment of nucleotide sequences.

### Phylogenetic estimation

The gene tree was reconstructed using two methods, maximum likelihood (ML) and Bayesian inference (BI). The ML phylogeny was estimated using PHYML v2.4.4 [42,43]; a general-time reversible model [44] was applied, with multiple substitution rates (six rate categories) and empirical base frequencies. The proportion of invariant sites and the gamma distribution parameters (α) were both optimised. An input tree was defined by neighbour-joining and robustness was assessed through 500 non-parametric bootstrap replicates [45]. AAT31Tc was assigned as the outgroup, based on its distance from other taxa in a neighbour-joining tree. AAT34Tc could not be satisfactorily aligned and so was not included in the analysis; this left a total taxon set of 93 individual sequences (derived from 35 loci).

The BI phylogeny was estimated using MrBayes v3.1.2 [46,47]. The substitution model was defined by the default settings with two exceptions: in the first analysis substitution rates were set to 'invgamma', and in a second analysis, a covarion model [48,49] was applied to assess the effect of coevolving sites on the estimation process. The optimal topology was determined using a Monte-Carlo Markov Chain (MCMC) process to search tree-space. 4 parallel MCMC chains were run for 1,000,000 generations, with a sample frequency of 100 generations and a burn-in of 5,000 generations. The output was checked for stationarity using Tracer v1.2.1 [50]; the burn-in was found to be sufficient to achieve stationary model parameters. AAT31Tc was again assigned as the outgroup and AAT34 TC was omitted.

### Reconciliation analysis

The phylogeny of a gene family transcends the history of an individual species, and genes can evolve independently of species phylogeny. Therefore, gene trees reflect, but are not identical to, species trees. By reconciling the differences between gene and species trees, it is possible to infer gene duplications that have occurred, as well as losses where genes were absent from genomic positions at which they were expected, given their presence in other species [51]. To determine the origins of AAT genes, the gene family phylogeny was reconciled with the simple species tree known for these organisms ([[*T. brucei, T. cruzi*], *L. major*]). Reconciliation analysis was executed by NOTUNG 2.1 [52], using default event costs. As this required a fully resolved, binary tree, the ML topology was applied. Putative duplications were classified as *transpositive*, i.e., resulting from transposition from one genomic position to another, or *tandem*, i.e., resulting from duplication in *cis *at the same position.

### Relative rate analysis

Phylogenetic trees contain a temporal component that may provide evidence of non-random changes in evolutionary rate along a branch. The phylogeny estimated here was split into 26 subclades to partition sequences into groups of close relatives, plus appropriate outgroups. These outgroups had to be robustly placed with the ingroup, not too distant to cause inaccurate measures of genetic distance (i.e., exposure to substitution saturation), but distant enough to offer a clear comparison between members of the ingroup. Each subclade was analysed using relative rate tests [53,54] with RRtree v1.1.11 [55]. The length of the branch leading to each sequence was estimated using the number of non-synonymous substitutions per non-synonymous site [56]. These values were compared with close relatives to identify those lineages that had experienced a significant change in evolutionary rate, since sharing an ancestor [57]. Synonymous sites were not used since these were saturated in places and often could not be accurately estimated.

### Assessment of concerted evolution

Concerted evolution occurs among repetitive sequences, such as gene family members, where distinct sequences within a genome become homogenised, losing the identity they should display towards orthologs in other genomes [58,39]. Concerted evolution should result in monophyly of all gene copies within a genome, relative to copies at the same position in other genomes. To assess its effect on tandem gene arrays, relevant loci in *T. brucei *were combined in a ML phylogenetic tree with homologs from the same genomic positions in close relatives *T. congolense, T. vivax *and *T. cruzi *(genome sequences retrieved from GeneDB), using the same procedure described for the gene family tree. Tandem gene arrays from *L. major *were similarly compared with homologs from *L. infantum *and *L. braziliensis*. The copy number of repetitive genes in the *T. cruzi *genome sequence is not well characterised, precluding analysis of this species. The significance of any monophyly identified in the phylogenetic trees was assessed using the SH test [59]; the likelihood of the observed topology was compared with the likelihoods of topologies forced to show monophyly by species (i.e., concerted evolution), or monophyly by position (i.e., retention of orthology).

### Assessment of gene conversion

Gene conversion occurs through non-homologous recombination between repetitive sequences, resulting in the partial or total homogenisation of multiple sequences. In the present context, it may affect gene family evolution by exchanging sequence motifs among family members within a genome, affecting their identities and phylogenetic relationships. To detect gene conversion events, sequence alignments of all sequences were prepared for each species individually. These were analysed within the RDP v2.0 platform [60] using the GENECONV program [61], which identifies strings of silent polymorphisms shared in sequence triplets that exceed chance expectations. Putative conversion events were inspected by eye and checked using a second method, SISCAN [62], which applies a phylogenetic profile approach to identify regions of an alignment that give a significantly different phylogenetic signal to neighbouring regions. Both GENECONV and SISCAN assess the significance of putative conversion events with permutation tests, creating 1000 randomized data sets to assess the frequency with which a given region of similarity occurs by chance.

## Supplementary Material

Additional File 1 Results of relative rates tests. Table showing significant (bold) and near-significant results of relative rates tests on various pairs of lineages, as applied to non-synonymous substitutions per non-synonymous site using RRtree. Click here for file
