# Supplementary material for: Origins of amino acid transporter loci in trypanosomatid parasites
Source: BMC Evol Biol. 2007 Feb 23;7:26. doi: 10.1186/1471-2148-7-26 (PMC1810246; doi:10.1186/1471-2148-7-26)
Supplement: Additional File 1 — Results of relative rates tests. Table showing significant (bold) and near-significant results of relative rates tests on various pairs of lineages, as applied to non-synonymous substitutions per non-synonymous site using RRtree. [file 1471-2148-7-26-S1.doc]

Results of relative rate tests: showing significant (bold) and near-significant results of relative rate tests on various pairs of lineages, as applied to non-synonymous substitutions per non-synonymous site.

| Outgroup | Lineage1 | Lineage2 | Non-synonymous sites: | | | | |  | |
| --- | --- | --- | --- | --- | --- | --- | --- | --- | --- |
|  |  |  | # | Ka1 | Ka2 | ΔKa | SD | Ratio | P |
|  |  |  |  |  |  |  |  |  |  |
| AAT30Tc | AAT1Tc | AAT1.4Lm | 810.2 | 0.4488 | 0.5150 | -0.0662 | 0.0310 | -2.1371 | **0.0326** |
| AAT30Tc | AAT23.1Lm | AAT1.4Lm | 800.4 | 0.4683 | 0.5150 | -0.0467 | 0.0247 | -1.8946 | 0.0581 |
| AAT30Tc | AAT1.2Lm | AAT1.4Lm | 800.4 | 0.4708 | 0.5150 | -0.0441 | 0.0246 | -1.7953 | 0.0726 |
| AAT30Tc | AAT1.3Lm | AAT1.4Lm | 800.4 | 0.4708 | 0.5150 | -0.0441 | 0.0246 | -1.7953 | 0.0726 |
| AAT18Lm | AAT33Tc | AAT24Lm | 768.9 | 0.6773 | 0.7469 | -0.0695 | 0.0372 | -1.8706 | 0.0614 |
| AAT18Lm | AAT33Tc | AAT5.1Tb | 747.4 | 0.6773 | 0.7599 | -0.0825 | 0.0446 | -1.8495 | 0.0644 |
| AAT18Lm | AAT33Tc | AAT5.2Tb | 749.4 | 0.6773 | 0.7555 | -0.0782 | 0.0439 | -1.7789 | 0.0753 |
| AAT18Lm | AAT33Tc | AAT5.3Tb | 745.8 | 0.6773 | 0.7546 | -0.0773 | 0.0439 | -1.7596 | 0.0785 |
| AAT18Lm | AAT33Tc | AAT5.4Tb | 749.4 | 0.6773 | 0.7555 | -0.0782 | 0.0439 | -1.7789 | 0.0753 |
| AAT8.1Lm | AAT4.4Tb | AAT10.2Tb | 202.4 | 0.4083 | 0.2881 | 0.1202 | 0.0553 | 2.1756 | **0.0296** |
| AAT8.1Lm | AAT4.6Tb | AAT10.2Tb | 202.4 | 0.4056 | 0.2881 | 0.1175 | 0.0542 | 2.1701 | **0.0300** |
| AAT8.1Lm | AAT4.2Tb | AAT10.2Tb | 202.5 | 0.4019 | 0.2881 | 0.1138 | 0.0525 | 2.1686 | **0.0301** |
| AAT8.1Lm | AAT4.4Tb | AAT7.1Tb | 205.6 | 0.4083 | 0.3015 | 0.1068 | 0.0504 | 2.1181 | **0.0342** |
| AAT8.1Lm | AAT4.4Tb | AAT7.8Tb | 202.7 | 0.4083 | 0.3007 | 0.1076 | 0.0511 | 2.1036 | **0.0354** |
| AAT8.1Lm | AAT4.6Tb | AAT7.1Tb | 205.6 | 0.4056 | 0.3015 | 0.1041 | 0.0502 | 2.0730 | **0.0382** |
| AAT8.1Lm | AAT4.2Tb | AAT7.1Tb | 205.7 | 0.4019 | 0.3015 | 0.1004 | 0.0486 | 2.0641 | **0.0390** |
| AAT8.1Lm | AAT4.6Tb | AAT7.8Tb | 202.7 | 0.4056 | 0.3007 | 0.1049 | 0.0509 | 2.0608 | **0.0393** |
| AAT8.1Lm | AAT4.2Tb | AAT7.8Tb | 202.7 | 0.4019 | 0.3007 | 0.1012 | 0.0494 | 2.0497 | **0.0404** |
| AAT8.1Lm | AAT4.4Tb | AAT7.10Tb | 202.4 | 0.4083 | 0.3018 | 0.1065 | 0.0531 | 2.0069 | **0.0448** |
| AAT8.1Lm | AAT4.6Tb | AAT7.10Tb | 202.4 | 0.4056 | 0.3018 | 0.1038 | 0.0528 | 1.9648 | **0.0494** |
| AAT8.1Lm | AAT4.2Tb | AAT7.10Tb | 202.5 | 0.4019 | 0.3018 | 0.1001 | 0.0513 | 1.9496 | 0.0512 |
| AAT8.1Lm | AAT4.4Tb | AAT7.6Tb | 202.9 | 0.4083 | 0.3074 | 0.1009 | 0.0519 | 1.9448 | 0.0518 |
| AAT8.1Lm | AAT4.6Tb | AAT7.6Tb | 202.9 | 0.4056 | 0.3074 | 0.0982 | 0.0517 | 1.8997 | 0.0575 |
| AAT8.1Lm | AAT4.2Tb | AAT7.6Tb | 203.0 | 0.4019 | 0.3074 | 0.0945 | 0.0502 | 1.8839 | 0.0596 |
| AAT8.1Lm | AAT10.1Tb | AAT10.2Tb | 202.3 | 0.3657 | 0.2881 | 0.0776 | 0.0414 | 1.8765 | 0.0606 |
| AAT8.1Lm | AAT4.4Tb | AAT7.2Tb | 208.8 | 0.4083 | 0.3115 | 0.0968 | 0.0524 | 1.8447 | 0.0651 |
| AAT8.1Lm | AAT4.6Tb | AAT7.2Tb | 208.8 | 0.4056 | 0.3115 | 0.0941 | 0.0513 | 1.8335 | 0.0667 |
| AAT8.1Lm | AAT2.2Tb | AAT10.2Tb | 204.4 | 0.3595 | 0.2881 | 0.0714 | 0.0401 | 1.7793 | 0.0752 |
| AAT8.1Lm | AAT2.3Tb | AAT10.2Tb | 204.4 | 0.3595 | 0.2881 | 0.0714 | 0.0401 | 1.7793 | 0.0752 |
| AAT8.1Lm | AAT2.4Tb | AAT10.2Tb | 204.4 | 0.3595 | 0.2881 | 0.0714 | 0.0401 | 1.7793 | 0.0752 |
| AAT8.1Lm | AAT4.2Tb | AAT7.2Tb | 208.9 | 0.4019 | 0.3115 | 0.0904 | 0.0512 | 1.7638 | 0.0778 |
| AAT28Lm | AAT14Lm | AAT14Tc | 677.0 | 1.2168 | 1.0607 | 0.1561 | 0.0842 | 1.8530 | 0.0639 |
| AAT28Lm | AAT14Tb | AAT14Tc | 653.5 | 1.2302 | 1.0607 | 0.1695 | 0.0695 | 2.4387 | **0.0147** |
| AAT21Lm | AAT15Tb | AAT15Lm | 780.5 | 0.8031 | 0.7164 | 0.0867 | 0.0434 | 1.9972 | **0.0458** |
| AAT33Tc | AAT16Tc | AAT18Lm | 744.3 | 0.3601 | 0.6734 | -0.3133 | 0.0443 | -7.0705 | **0.0000** |
| AAT33Tc | AAT18Lm | AAT16Lm | 744.2 | 0.6734 | 0.3646 | 0.3088 | 0.0446 | 6.9211 | **0.0000** |
| AAT33Tc | AAT18Lm | AAT16.1Tb | 731.5 | 0.6734 | 0.3875 | 0.2859 | 0.0444 | 6.4377 | **0.0000** |
| AAT33Tc | AAT18Lm | AAT16.2Tb | 731.4 | 0.6734 | 0.3837 | 0.2897 | 0.0444 | 6.5193 | **0.0000** |
| AAT8.2Lm | AAT17.1Tb | AAT17.2Tb | 791.6 | 0.4838 | 0.4377 | 0.0461 | 0.0163 | 2.8256 | **0.0047** |
| AAT8.2Lm | AAT17.1Tb | AAT17Tc | 784.5 | 0.4838 | 0.4259 | 0.0579 | 0.0277 | 2.0879 | **0.0368** |
| AAT1Tc | AAT19Lm | AAT30Tc | 818.5 | 0.5055 | 0.4488 | 0.0567 | 0.0284 | 1.9957 | **0.0460** |
| AAT12Tc | AAT32Tc | AAT36Tc | 817.9 | 0.7393 | 0.7516 | -0.0123 | 0.0062 | -1.9835 | **0.0473** |
| AAT3Tb | AAT35Tc | AAT22Lm | 784.7 | 0.4706 | 0.5260 | -0.0553 | 0.0299 | -1.8522 | 0.0640 |
| AAT28Lm | AAT26Tc | AAT27.2Lm | 775.6 | 0.9074 | 1.0130 | -0.1056 | 0.0590 | -1.7904 | 0.0734 |

The lineage showing greater evolutionary change is shaded.
